# Bio-Inspired Electricity Storage Alternatives to Support Massive Demand-Side Energy Generation: A Review of Applications at Building Scale

**DOI:** 10.3390/biomimetics6030051

**Published:** 2021-08-26

**Authors:** Alisson Dodón, Vanessa Quintero, Miguel Chen Austin, Dafni Mora

**Affiliations:** 1 Research Group in Energy and Comfort in Bioclimatic Buildings, Faculty of Mechanical Engineering, Universidad Tecnológica de Panamá, Panama City 0819, Panama; alisson.delarosa@utp.ac.pa (A.D.); vanessa.quintero1@utp.ac.pa (V.Q.); miguel.chen@utp.ac.pa (M.C.A.); 2 Faculty of Electrical Engineering, Universidad Tecnológica de Panamá, Panama City 0819, Panama; 3 Centro de Estudios Multidisciplinarios en Ciencias, Ingeniería y Tecnología (CEMCIT-AIP), Panama City 0819, Panama

**Keywords:** biomimicry, buildings, electricity, energy, storage systems

## Abstract

This work has its origin in the growing demands of energy regulations to meet future local targets and to propose a global implementation framework. A literature review related to conventional electrical energy storage systems has been carried out, presenting different cases analyzed at building scale to deepen in nature-inspired processes that propose reductions in environmental impact and present improvements in these storage devices. The use of batteries, especially lithium-ion batteries, is the most prominent among the electrical storage applications; however, improvements have been proposed through hydrogen batteries or the implementation of more environmentally friendly materials to manufacture the electrodes. In this sense, oriented to creating systems designed to protect the environment, important advances have been made in the development of storage systems based on biomimetic strategies. The latter range from the generation of energy through the respiratory processes of microorganisms to the recreation of the generation, storage, and release of energy using the thermoelectric and thermoregulatory characteristics of some insects. These facts show that the trend in research towards improving existing systems continues but reinforces the idea that new solutions must be environmentally friendly, so there is still a long way to improving the processes established thus far.

## 1. Introduction

Globally, increasing demands are identified within their energy regulations, and in order to meet future goals, renewable energy systems have been implemented to supply the amount of energy required in a timely and environmentally friendly manner. These renewable energy sources need a storage system to supply the demand when the source cannot supply electricity. Although these systems already exist, they are not entirely “green” for the environment.

Energy storage systems (ESS) convert electrical energy from power systems into a form in which it can be stored and subsequently transformed into electrical energy when required by the consumer. Energy systems play a key role in collecting energy from various sources and converting it into forms of energy needed for various applications in various sectors such as utilities, industry, transportation, and construction [1]. Energy storage can provide several advantages for energy systems such as allowing higher penetration of renewable energy, reducing energy losses in the distribution system, increased reliability and customer satisfaction, better economic performance, among other factors. Even energy storage is of great importance in power systems as it allows load leveling, peak shaving, frequency regulation, damping of oscillations, and improvements in power quality and reliability.

The operation of energy storage systems, according to [2], is categorized into:

- Charging period: the power grid is used during off-peak intervals when electric power is at a lower cost.
- Discharge period: at peak demand time, the stored energy is used. During this period, the grid has a higher cost, so the use of distributed generation makes it more economical. Consequently, storage systems are applied to reduce or eliminate the uncertainties of distributed generation.

In terms of capacity, these systems are divided into:

- Large-scale storage (GW): reversible hydro (pumped), thermal storage.
- Grid and generation asset storage (MW): cells, batteries, condensers, superconductors, flywheels.
- End-user storage (kW): batteries, superconductors, flywheels.

Before, this originated the present qualitative and descriptive study to highlight all those advantages and disadvantages, among other technical aspects with the different conventional electrical energy storage systems. An extensive literature review of the different conventional electrical storage systems was carried out, together with an application at different scales, such as residential and commercial, to compare them.

Taking these factors into account, we searched for mechanisms inspired by nature that have the capacity to generate and store electrical energy in order to have a set of pinnacles that serve as a basis for the design of an electrical storage system that reduces the environmental impact. Among different studies reviewed, the criteria, principles, and characteristics of how such biomimetic approaches could be used in conventional systems are presented.

## 2. Materials and Methods

This research is based on developing a descriptive and qualitative methodology of both the different types of electrical energy storage systems and the organisms (“pinnacles”) that generate or accumulate electricity naturally.

The EBSCO, MDPI, Elsevier, and Google Scholar databases were used to investigate conventional storage systems, while, for the part of the mechanisms inspired by nature, databases such as Springer US, Journal of Biological Engineering, and Ask Nature were used.

**Figure 1:**
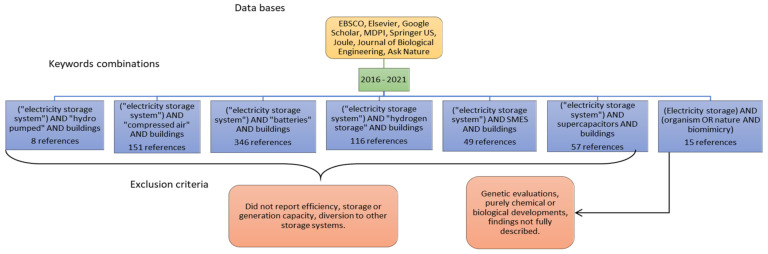
Literature search strategy. Own elaboration.

The Boolean operators (AND, OR, NOT) combined with the keywords electrical storage systems, buildings, nature, and biomimicry were applied as a search strategy (Figure 1). The search period was based on the last five years (2016–2021).

For the conventional section, there were no problems since there were indeed several studies related to electrical energy storage applied to both residential and commercial buildings; even so, those articles that involved other types of storage such as thermal storage were discarded. Likewise, with the section on mechanisms inspired by nature, articles in which the topic was more focused on aspects such as genetics of living beings, totally chemical or biological approaches were discarded.

## 3. Conventional Electricity Storage Strategies: Concept and Applications at Building Scale

Energy storage systems have been a fundamental piece for renewable sources since they can supply the energy demands when the system cannot. In this section, different study cases are presented applying each configuration at a building scale in different types of climates. At the end of this section a summary of the studies encountered including advantages and disadvantages of each conventional electricity storage systems is presented.

### 3.1. Pumped-Hydro Energy Storage (PHES)

Pumped-hydro energy storage (PHES) has two reservoirs or basins (one high and one low) connected via tunnels and shafts through which water can be passed from one point to the other. Hydro turbines, pumps, and valves are found to control water’s flow from one reservoir to the other and generate electricity when necessary [3]. In this type of system, electrical energy accumulates electricity in hydraulic potential energy form by means of an electric pump that carries water from a lower level to a higher-level during hours of low energy demand. The turbine is connected to an electricity generator. The inlet flow of water can be controlled using gates to allow a variable power output. In addition, variable-speed drives can be used to provide regulation during the charging state [1].

Today’s metropolitan cities have a natural height difference for potential energy, an advantage that has not been fully exploited. This gives rise to improving the use of renewable sources in a photovoltaic configuration co-connected to the grid together with hydro-pump storage. For example, a study was conducted in 2020 (Shanghai, China) based on the city’s electricity pricing policies to develop the feasibility of renewable technology applied in residential villas and apartments [4].

**Figure 2:**
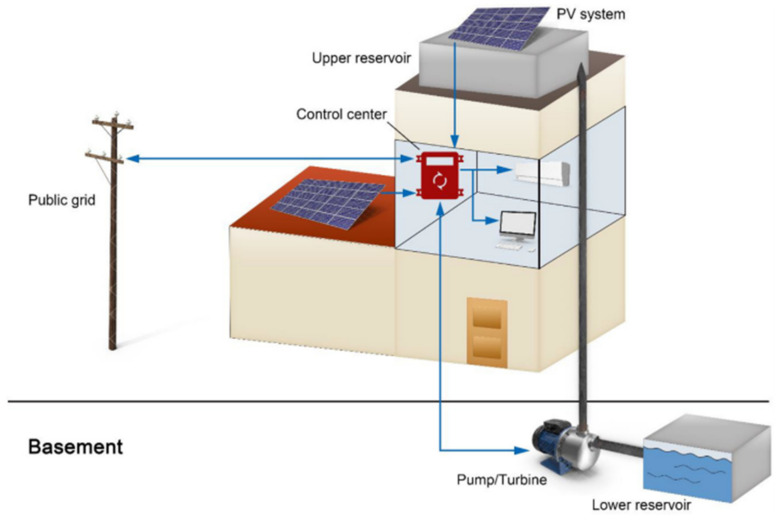
Configuration of the PV arrangement with the hydro-pump system [4]. No changes were made to the original figure.

Figure 2 illustrates the array of this system equipped with photovoltaic panels for its generation and the pumped-hydro energy storage. Its control, apart from acting as an inverter, also behaves as a control for electricity distribution. The study focuses on the fact that the pumping system can store excess energy from the photovoltaic (PV) system by pumping the water to the upper reservoir, where it will be used when it does not meet the required demand [4].

The installed capacity of the whole system is 5 kW, according to Shanghai applications. The villas are three stories with a height drop of 13 m, an upper reservoir with a volume of 100 m^3^ was installed, the pumping coefficient of the storage system was 24 m^3^/kW, and that of the generation turbine, 0.0319 kW h/m^3^. For this case, the consumption of the villa load is defined as twice the electricity consumption of an apartment household. The apartment consisted of seven floors with a height drop of 22 m, the same volume was used for the upper reservoir of the system, and the pumping and turbine coefficients were 14.19 m^3^/kW h y 0.0539 kW h/m^3^, respectively.

As a result, it was shown that the system is more viable for an apartment building than for the villas since, comparing the state of loads of both, the pumping system in a villa only operated in a so-read period (10 a.m.–6 p.m.); after this time, the water storage is null so it does not provide energy to the residents, In addition, the villa depends on the national grid after 7:00 p.m., while the case of the apartments reflected a longer duration to supply energy (10:00 a.m.–10:00 p.m.) and its storage maintained more than 10–30% of the water volume during the days demonstrating that the pumping system has enough potential to ensure the generation of electricity. However, for both cases, the power generated from the PV systems on cloudy or rainy days was ineffective for water storage in the reservoir. The self-consumption and self-sufficiency rates for the apartment were 59.69% and 76.47%, while, for the villa, they were 66.25% and 45.13%, respectively, concluding that, in the energy balance, the results show that the apartment absorbs more surplus energy from the PV system rather than selling it to the grid. In addition, the system is able to feed the appliance load compared to the villa by reducing the proportion of energy supplied directly from the grid [4].

### 3.2. Compressed Air Energy Storage (CAES)

In compressed air energy storage systems, also known as compressed air energy storage (CAES) systems, the air is compressed and stored in an underground reservoir as long as there is excess energy. Usually, underground reservoirs are caverns drilled in salt or rock formations, abandoned mines, or existing cavities of minerals or aquifers. If energy is needed, stored air will expand to a turbine which generates electricity [1,5].

**Figure 3:**
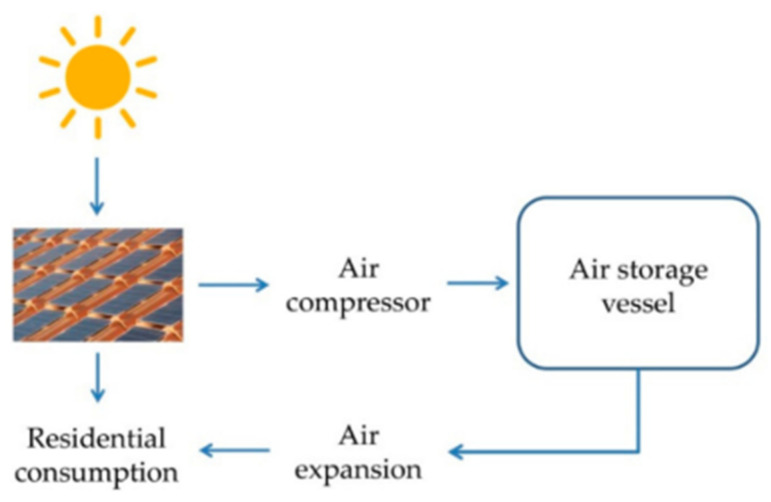
Compressed air energy storage system (CAES system) connected with the residential photovoltaic (PV) plant [6]. No changes were made to the original figure.

In 2018, a study of a prototype system was presented, which consisted of the use of a photovoltaic array coupled with CAES to compress air and, when expanded, generate the demanded electrical energy. This small-scale system was located in an unoccupied basement of a building in Cittá di Castello (Perugia, Italy). The system associated with the residential photovoltaic plant is shown in Figure 3. The study carried out three scenarios for its energy storage: small-scale CAES using 30 bar compressor pressure, small-scale CAES at 225 bar, and, finally, a lead-acid battery in order to make a comparison between them [6].

The photovoltaic energy production used for air compression is between 26.9 kWh/day in terms of average energy consumption of a residential building on a summer day, and a compressor with a flow rate of 4 Nm^3^/h was considered for the results below:

- With a pressure of 30 bar, the compressor absorbed 8702 kWh of excess PV energy in a 1.7 m^3^ vessel. Expanding through the turbine generated 1008 kWh in 38 min which covered 21.9% of the residential energy demand.
- At a pressure of 225 bar, 96% of the excess PV energy was absorbed within a 0.25 m^3^ enclosure. The expansion generated 1273 kWh, which covered 26% of the residential energy demand.
- Using the lead-acid battery with 80% charge efficiency was able to cover 100% of the total demand, which was equivalent to 4.6 kWh, demonstrating that, in terms of overall efficiency, the CAES system is less than any electrochemical battery system.

### 3.3. Flywheel Energy Storage System (FESS)

**Figure 4:**
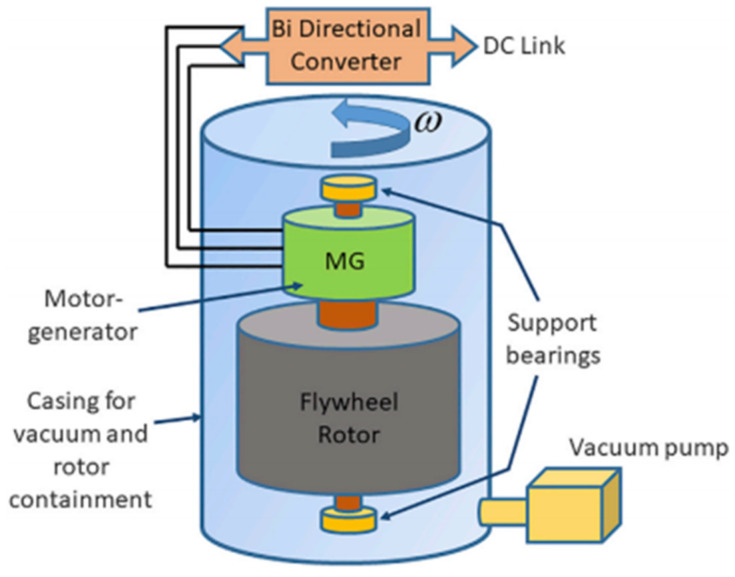
Components of a flywheel system for electrical storage based on [8] reproduced by [10].

This type of system consists of a mechanical energy storage form that is suitable for achieving the smooth operation of machines and providing high power and energy density [1]. A flywheel uses a rotating mass to store energy which is held in the kinetic energy of rotation of the rotor. The amount of stored energy is proportional to the moment of inertia of the rotor; hence, increasing rotational speed will increase storage capacity, but higher speeds offer a more efficient way of raising capacity. However, high speeds can make severe demands on the materials used in flywheel construction [7]. This kinetic energy is transferred in and out of the flywheel using an electrical machine that acts as a generator or motor depending on whether the system is in charge or in discharge mode. Generally, permanent magnet machines are common for this type of system due to their high efficiencies, high densities, and low rotor losses [8]. Flywheels convert the electrical energy surplus into motion in a high-speed rotating disk that is connected to an electric motor [9]. The main components of the FESS systems are shown in Figure 4.

### 3.4. Battery Energy Storage System (BESS)

Electrical energy can be stored electrochemically within batteries or capacitors. Batteries are the most used devices for electricity storage purposes. They can react instantaneously to changes in energy demand, and the type of cells used together to generate electricity can deliver and absorb energy quickly. During the chemical reaction of batteries, 80% or more of the energy is released to convert it into electrical energy, but this percentage varies with the type of battery, discharge rate, among other aspects [11].

Currently, there are several types of batteries, such as lithium batteries, including lithium-ion and lithium hydride batteries which represent the most popular battery type among consumer electronic devices due to their low weight, low self-discharge, high energy density, and long cycle life. Lead-acid batteries were one of the first batteries to be developed and were used for load leveling in some power distribution systems. There were also nickel-cadmium batteries that had high energy densities and were lighter than lead-acid batteries, and were even used in cell phones and laptops; however, they were replaced by lithium-ion batteries [11]. Continuing with the nickel battery family, nickel-metal hydride batteries function as another substitute for nickel-cadmium batteries due to their high energy densities and the absence of toxic metals representing less impact on the environment. Unlike lithium-ion batteries, this type of battery has a longer life cycle and better price [12]. Table 1 presents a comparison between the characteristics of the different types of batteries in terms of nominal voltage, life cycle, energy density, and self-discharge.

In 2016, a comparison was made between different types of batteries (Lead Acid, NaNiCl, Lithium) for energy storage together with a photovoltaic configuration for residential buildings in Sweden where the average peak consumption was in February with a value of 2419 kWh and average production of 5.20 kWh while the lowest consumption was in June with average consumption and production of 1224 kWh and 0.51 kWh, respectively. Among the battery comparisons, the lithium battery with a modular capacity of 7 kWh and efficiency of 92% was highlighted, concluding that this type of battery provides a high self-sufficiency rate, which made it quite convenient for the case study due to its seasonal changes, storing excess energy in summer for consumption in Winter [14].

**Figure 5:**
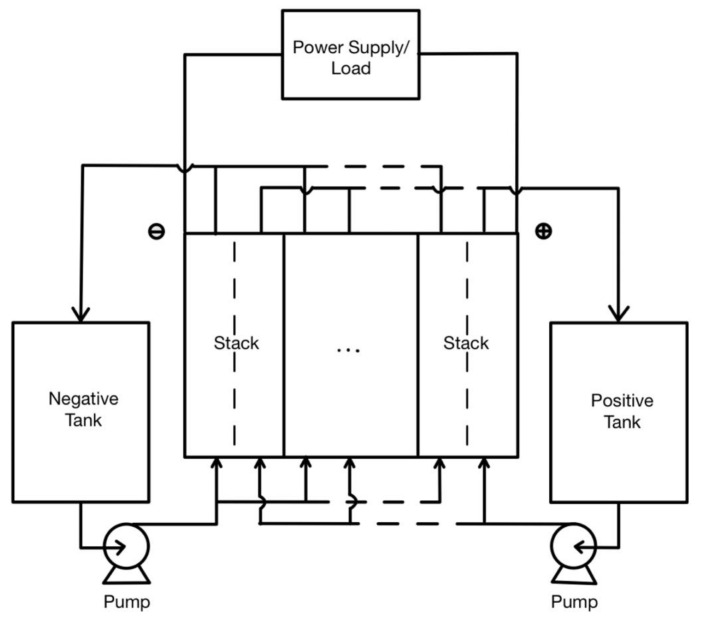
Basic configuration of the vanadium-redox system which consists of electrolyte tanks (positive and negative), stacks, endplates, and pumps [15]. No changes were made to the original figure.

Vanadium-redox batteries (VRBs) are also available in the market since, thanks to their attractive characteristics in terms of a long-life cycle, high energy efficiency, and low maintenance cost, the use of these batteries has been employed in the residential sector together with photovoltaic generation, and it has proven to be cost-effective to date. In 2016, the authors in [15] proposed an optimal sizing method for vanadium redox battery systems in residential-scale applications considering aspects of cost, battery efficiency, time-varying electricity price, solar feed-in tariff, user consumption, and PV profiles. It provided guidance for capital cost calculation, maintenance of the system itself, and an approach to charge/discharge efficiency evaluation of these batteries. These types of systems are based on the scheme in Figure 5.

Recently, new electrode and electrolyte materials have been developed to improve the advantages, cost, and safety of these devices. Batteries and supercapacitors are usually compared to each other, with batteries having better storage capacity by more than 30 times the charge per unit mass than supercapacitors; however, supercapacitors are able to deliver up to thousands of times the power of a battery of the same mass because they accumulate energy by adsorption reactions on the surface of the electrode material [1].

### 3.5. Supercapacitor Energy Storage System

Capacitors are electronic devices that store electrical energy directly in the form of electrostatic charge. The simple arrangement of a capacitor consists of two metal plates separated by a small air gap. When voltage is applied across the device, the plates become statically charged; when the voltage is removed, the charge remains until a short circuit between plates occurs. The amount of charge accumulated on each plate creates an electric field that balances the charge generated by the voltage [16].

A conventional capacitor operates in the order of millifarads. A supercapacitor stores energy in the order of farads and more, whose fundamental characteristic is denoted by its ability to charge and discharge in seconds or less time. One application of this device is the electric car since the charges and discharges of a supercapacitor allow the car to recover part of its autonomy faster and more efficiently [17]. On the other hand, they have limited storage capacity. Current supercapacitors have a storage energy density of about one-tenth that of a lithium-ion battery. The voltage of these devices drops as their charge also drops, while that of a battery remains about the same for most of its de-charging cycle. This affects how each can be used [16].

There are three types of supercapacitors according to [17] as these are classified according to the composition of the dielectric material or conductor used:

- Electrochemical double-layer supercapacitors (EDLC): this electrochemical double-layer capacitor uses its two charge layers when a voltage is applied to an electrode (made of highly porous carbon) immersed in an electrolytic substance. Its slight charge separation generates capacitances on the order of 40–60 F/cm^3^. Unlike conventional capacitors, the energy required to enter the breakdown field is very high and is usually calculated in V/cm.
- Supercapacitors with pseudocapacitance: this type of supercapacitors store charges in a faradic form rather than electrostatical by means of a charge transfer between an electrode and an electrolyte. They can be composed of polymer materials or metal-oxide compounds and, therefore, have different costs, conductivity, pseudocapacitance, and application characteristics. A supercapacitor based on metal-oxide composites has a high energy storage density and therefore is very similar to EDLC capacitors.
- Hybrid supercapacitors: they are a mixture of EDLC supercapacitors together with pseudocapacitors since this system combines both faradic and non-faradic processes to store the charge. Currently, several tests have been carried out with different elements such as ruthenium dioxide (RuO_2_), cobalt oxide (Co_3_O_4_), nickel oxide (NiO), vanadium oxide (V_2_O_5_), nickel hydroxide (Ni(OH)_2_, and manganese oxide (MnO_2_), the latter having the best qualities since it is abundant and friendly material on Earth. This type of supercapacitor is widely used in battery systems to improve their efficiency.

### 3.6. Superconducting Magnetic Energy Storage (SMES)

To achieve magnetic superconducting energy storage (SMES), a large superconducting coil can be used with almost no electrical resistance near absolute zero temperature and yet is capable of storing electrical energy within the magnetic field generated by direct current flowing through the field. SMES coils present large amounts of energy instantaneously and upon discharge, as well as an unlimited number of charge and discharge cycles with high efficiencies. Their energy discharge capacity is less than 100 ms, which presents a faster response time than batteries; however, the system requires constant cooling [18]. The main parameters for SMES design that could affect its storage are coil configuration, energy capacity, and operating temperature [1].

Some of the applications that include SMES are load leveling, system stability, voltage stability, frequency regulation, transmission capacity improvement, power quality improvement, automatic generation control, and uninterruptible power supply [1].

According to [18], SMES systems can stabilize the power grid by providing power quality to consumers even though such systems are costly. The same comprises distributed generation (DG) structures connected to the grid. The power generation plant, the conversion, and the storage unit are the main components of a commercial distributed generation facility. The conversion and storage components consist of an electrolyzer, fuel cell, tanks capable of controlling the rapid variations of electrical power, and its sudden demands from consumers. Resistance losses in SMES after its charging period are almost zero due to its superconducting coil. The cooling mechanism serves to keep the temperature of the superconducting coil below its critical value, such as Niobium–Titanium (NbTi), a superconducting material used for coils and liquid helium or superfluid coolants whose temperature is around 4.2 K to cool the system. SMES can release quantum energy during their discharge momentum to the power grid in fractions of milliseconds. Through a SWOT analysis, it is concluded that this new technology has many strengths: high energy capacity, stability, quality, fast response, and high stored efficiency without high risk of environmental impact. On the other hand, it has weaknesses as it is a system that demands high constant cooling, high-cost materials for its manufacture, high operating and maintenance costs, among other factors [18].

### 3.7. Hydrogen Energy Storage

Unlike other energy storage systems, that comprised of hydrogen offers a wide range of applications that can be used in various ways. The gas is attractive because of its low-carbon energy source and therefore does not generate carbon dioxide emissions during use. This reason is what makes hydrogen energy storage a high potential for energy storage.

Today, hydrogen is produced chemically from fossil fuels by electrolysis of water since water is a major component of the Earth; another feasible alternative is using renewable energy sources with a surplus of energy to produce hydrogen, which can be used in different applications.

Its principle is based on using the excess electricity produced by renewable sources to store it in the form of hydrogen, and, when an energy demand arises, the reserved hydrogen is used as a fuel in power plants [19]. Their systems are composed in their production of hydrogen by excess electricity by electrolysis, storage of the pro-produced hydrogen, and conversion of this stored element back to electricity in a controlled time [20].

Hydrogen storage represents a challenge for automotive applications. Hydrogen has a characteristically low energy density by volume, unlike other fuels such as petroleum and diesel. In addition, hydrogen is the lightest element of all and the most difficult to liquefy compared to methane and propane.

Fuel cells are low energy density devices like batteries that are capable of converting chemical energy into electricity. These cells show efficiencies around 70–80%, while, in some power plants, they reach efficiencies of 60%. Fuel cells use oxygen and hydrogen; these can be combined with super capacitors to increase their energy densities [1]. Photovoltaic and other solar systems depend on solar radiation and ambient temperature, wind turbines depend on wind direction, and hydroelectric plants depend on river flow. However, there are cases where the aforementioned renewable sources cannot provide the required amount of electricity, so the use of fuel cells becomes a good solution to the aforementioned problem because the cells are not dependent on weather conditions [21].

**Figure 6:**
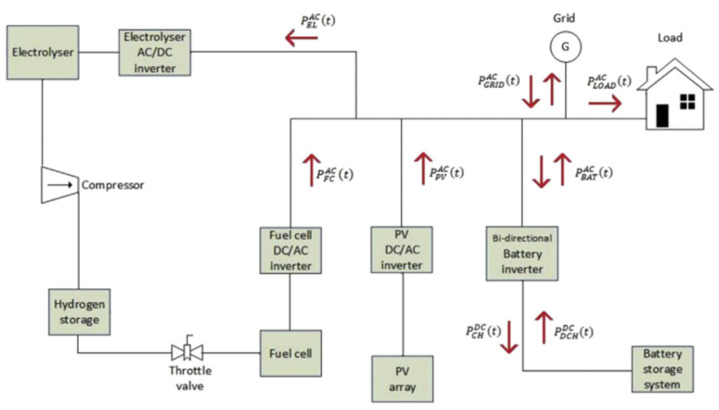
Scheme of the system configuration, fuel cells, and battery storage system used on [21]. No changes were made to the original figure.

Moreover, it has been proposed to use photovoltaic systems together with hydrogen-based fuel cells as they present a great opportunity to achieve self-sufficiency in electrical energy. In 2020, the proposal was developed by adding a battery storage system for a pilot building located in Slovenska Bristica, Slovenia. The system consists of photovoltaic modules placed on the roof of the building and has energy storage with lithium-ion batteries and an inverter. It is connected to the grid. Figure 6 shows the configuration of the system developed. Employing energy equations, aspects such as the balance of a hybrid system connected to the grid of the photovoltaic system and fuel cells with battery storage and its consumption through time “t” and the efficiencies of the batteries, hydrogen cells, inverter, and inverter electrolyzer were calculated. By means of modeling, the charging and discharging of the batteries were considered. With the fuel cell output, the system can operate when there is no sun exposure or when the batteries are not in optimal function. The hydrogen is stored in a tank, and, when it passes into the cell, it is recombined with oxygen, and electricity is generated [21].

For hydrogen production, the excess energy is used with electrolysis instead of extracting it from the grid. The study was carried out during one year where 202 days were of higher energy production than consumption, 162 days consumption was higher than production, and one day where consumption and production were equal.

As a result, the self-sufficiency of the fuel cell hybrid photovoltaic system was around 62.13%, which shows that it is not possible to complete the self-sufficiency of the pilot system. The hydrogen shortage was 144.24 kg. To achieve the desired self-sufficiency would require a larger photovoltaic system which would fit the correct dimensions and achieve the desired goal; even so, it would require an even larger hydrogen tank presenting high initial costs for its implementation. Battery storage is very effective for summer time, but, for winter time, hydrogen cells meet the shortcomings of conventional batteries (Table 2) [21].

## 4. Bio-Inspired Electricity Storage Strategies

**Figure 7:**
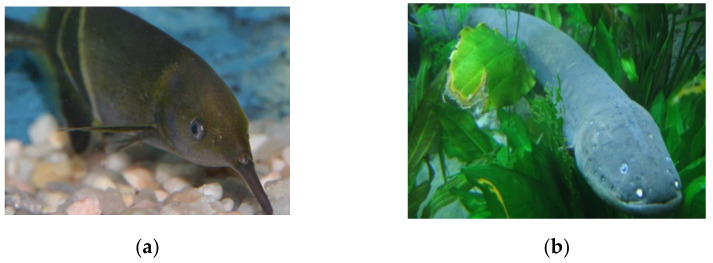
Fishes that can generate electrical energy in their bodies to search for food and navigate thorugh their habitat (**a**) Elephantnose fish (“*Gnathonemus petersii*”) [24] and (**b**) Electric eel (“*Electrophorus electricus*”) [25]. No changes were made to the original figures.

Nature is the principal source of life for many living beings. Therefore, it is interesting to visualize different behaviors, ecosystems, and anatomic aspects that can be useful as an inspiration to improve or create many new technologies. Members of both the animal and plant kingdoms exist because of energy—whether this comes from the sun stored in the form of sugars in plants, or from ingested food that is stored as fat in some animals. Within the animal or plant, energy is transferred at the electron level. A battery works in a similar way as the electricity is both taken in and discharged via the battery electrodes [23]. For instance, species such as electrical fishes are animals that manage to generate a certain amount of electrical energy through their bodies; they have certain points on their body’s special arrays similar to voltaic batteries, which allows these animals to produce an electrical discharge. These arrays are usually found in areas such as from the back to the belly, lateral parts of the fish’s body, tails, and sometimes almost cover the whole body. Figure 7 illustrates examples of this kind of fishes.

In the following subsection, a review of different organisms or pinnacles was made where each of them is related to energy storage or electricity generation. Table 3 presents the principal characteristics, mechanisms, and principles of each pinnacle.

### 4.1. Energy Storage, Photosyntesis

Photosynthesis is a biological mechanism that serves as an inspiration for the field of energy storage. Globally, it is estimated that photosynthetic organisms absorb an average of about 4000 EJ/year (130 TW) of sunlight. This capture is equivalent to 6.5 times the current global primary energy consumption of about 20 TW. Even so, photosynthesis is not perfect; it extracts carbon from the atmosphere at an average annual rate of 1 to 2 × 10^18^ CO_2_ molecules/m^2^s, which is 25 to 70 times less than the maximum possible rate of carbon absorption from the atmosphere of 5 a 7 × 10^19^ CO_2_ molecules/m^2^s. The overall and average annual efficiency of photosynthesis is between 0.25% and 1%, with the best efficiencies seen in the field at 2.4% for C3 plants (three carbon pathways), 3.4% for C4 plants (four carbon pathways), and 3% for algae grown in bubbled photobioreactors. The inefficiency of photosynthesis is because everything occurs within the same cell. Several alternatives have been developed to improve this aspect where photosynthesis is reconfigured by spatially separating each of the tasks performed within a photosynthetic organism and replacing some of them with a non-biological equivalent. These schemes have been termed “microbial electrosynthesis” or “rewired carbon fixation” by [26] with the object to capture and store solar energy from biofuels with higher efficiencies than photosynthesis; however, this separation allows storage of any electrical source.

**Figure 8:**
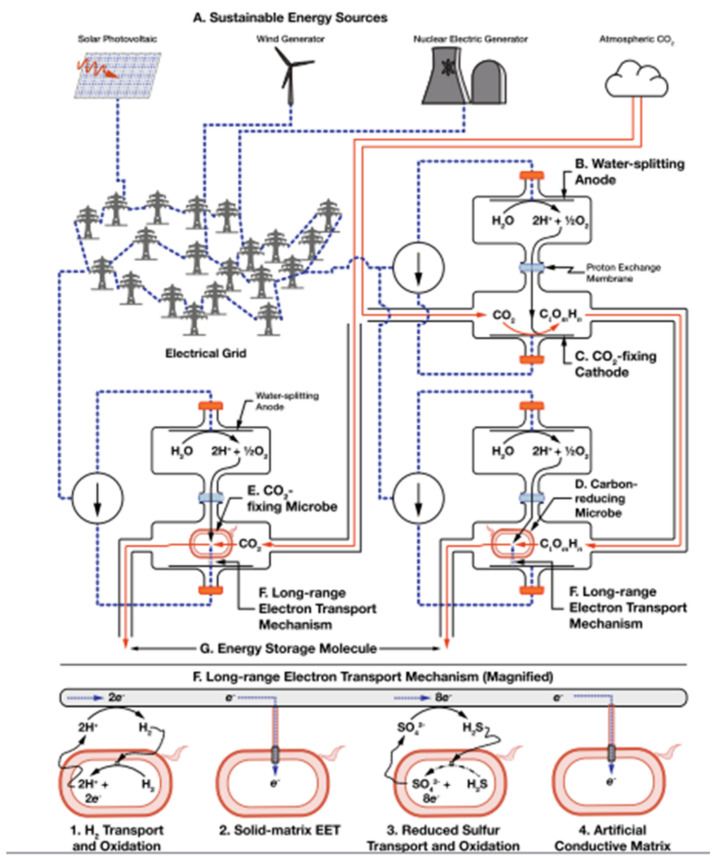
Fixation rewiring system consists of: (**A**) sustainable energy capture, (**B**) water splitting, (**C**) electrochemical CO_2_ fixation, (**D**) additional biological reduction (**E**) or biological CO_2_ fixation, (**F**) long-range electron transport to biological metabolism, and (**G**) synthesis of energy storage molecules [26]. No changes were made to the original figure.

From the configuration (Figure 8), two mechanisms for long-range electron transport and capture are highlighted: hydrogen transport to hydrogen oxidizing microbes and solid matrix extracellular electron transfer (SmEET) enabled by electroactive microbes. These microbes (Geobactor sulfurreducens, Sporomusa ovata, Ralstonia eutropha) are genetically engineered. In the same way, sulfur transport is developed along with its oxidation [26].

These biological advances in microorganism systems are becoming evolutionary tools in developing synthetic enzymes, autotrophic metabolisms, and self-assembling and self-repairing biological nanostructures, the latter being very useful in renewable energy systems [26].

### 4.2. Battery Electrode Materials

Lignin is a biopolymer abundant in the soil which is extracted from trees. This material is characterized as an important structural material in the supporting tissues of plants, some algae, and insects. Lignin has quinone as a substructure, a polymer of interest for energy storage through oxide-reduction (redox) reactions by which protons and electrons are absorbed and released. There are obstacles such as: short life cycle, low cyclic efficiency, and high self-discharge rate. The problem with using lignin is that the electrodes tend to degrade in the electrolytes.

The Venus flytrap has characteristic leaves divided into two movable halves. Once the prey lands inside the open leaves, these halves are closed, imprisoning the prey inside the plant. As a biomimetic strategy, the capture form of this living creature is mimicked by means of a reconfigurable graphene cage. This confines the lignin within the electrode to prevent dissolution while acting as a three-dimensional current collector to provide efficient electron transport pathways during the electrochemical reaction. This bio-inspired design exhibited 88% capacitive retention for 15,000 cycles and 211 F/g layer-cytance at a current of 1.0 A/g. This study demonstrates the effectiveness and solves the problem of the cyclic lifetime of the electrochemically lignin-based species to make use of this material as effective, economical, and renewable [27].

### 4.3. Energy Production, Anatomy of Plants

**Figure 9:**
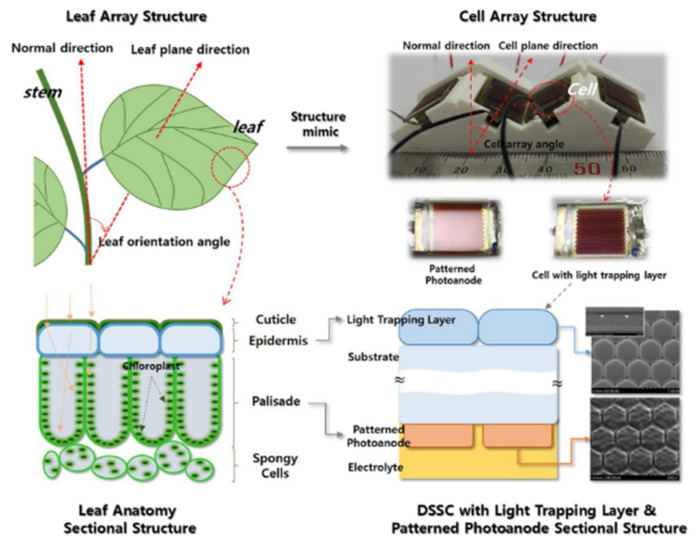
Anatomical structure of plant leaves as a basis for dye-sensitized solar cell (DSSC) configuration [28]. No changes were made to the original figure.

Plants are the most efficient light scavengers in existence. Their behavior has opened doors for creating new photovoltaic cells that can be applied in urban systems. Plants have the advantage of adapting to any environment. The orientation of their leaves is generally towards the light and not in a vertical position because the crown of the leaf surface is wide, and the inclination limits the light needed for photosynthesis, which makes this structure optimal for the collection of indirect and scattered illumination. Photosynthesis is a slow chain reaction. The leaf anatomy balances the number of photon incidences to those consumed by photosynthesis to maximize its efficient collection. Figure 9 shows the analogy of leaf anatomy used to recreate the solar cell arrangement [28].

Mimicking plant leaves’ structure and anatomy, one study created a light-capturing layer on top of the cells that mimic the epidermis. For the palisade structure, microscale photoanodes were used. One of the findings of the study was that, using 2D ray tracing, the trapping layer absorbed the incident light omnidirectionally and distributed it homogeneously across the photo-anodes. The current densities and light distribution were analyzed using the finite element method (FEM). The light-trapping layer and photo-anode tracing doubled the efficient conversion of dye-sensitized solar cells (DSSCs) from 4% to 8% by modifying the light distribution and improving the charge collection efficiency. Taking this study to the module scale, it was shown that DSSCs are much more efficient when illuminating the cells obliquely. To improve the efficiency of the system module, they connected in clusters of four DSSCs in parallel, mimicking the way plant leaf crowns exhibit a phyllotactic arrangement. The electrical power output improved by almost 55% by introducing the light-trapping layer designed in the study compared to the cells used in conventional designs [28].

### 4.4. Energy Generation by Respiratory Reactions of Microorganisms

From 2003 until today, BioGenerators have been developed, which are bio-electrochemical systems that use the respiratory reaction of a microorganism (*Leptospirillum ferriphilum*) as an electron collector for the generation of electrical energy. It is also a negative emitter of CO_2_ consumed from the atmosphere as part of electricity generation. In 2017, three BioGenerators were built whose bioreactors varied in volume, dimensions, and fabrication material, but with the same culture of microorganisms. Their electro-chemical cells were built in different sizes, but the material for the anodes, cathodes, and bipolar plate were the same (graphene). Two types of membranes were used for the device membranes: cation exchange membrane and polyvinyl alcohol-based membrane. The microbial culture was obtained from acid mine drainage samples from four sites (USA, Spain, Bulgaria, Finland). Air was injected into the bioreactors to supply the microorganisms with oxygen and carbon dioxide [20].

The biological oxidation of ferrous ions by *Leptospirillum ferriphilum* is essential for the operation of this device since these ions were used as cathode electrons in the electrochemical cell where the anode electron donor was hydrogen gas. After the main biological and electrochemical reactions, the microorganisms act as biocatalysts increasing the rate of oxygen reduction. It is worth mentioning that these microorganisms were treated by analytical and genetic engineering techniques. *Leptospirillum ferriphilum* as autotrophic organisms (producing their food) use CO_2_ from the atmosphere as a sole carbon source, making the BioGenerators commercially viable as CO_2_-negative systems. For this case, the ferrous ions did not need electro-catalysts based on precious metals since the cathode was made of carbon felt, which makes it more economical than a conventional proton exchange membrane (PEM), for the oxidation of the hydrogen-based anode, PEM fuel cells were used with a quantity of black platinized carbon. As a result, a current density of 1.35 A/cm^2^ and a maximum energy density of 1800 W/m^2^ were achieved. The cell voltage was 650–800 mV with a voltage efficiency of 46–57%. Its overall efficiency reached 70%. These Bio-Generators have gradually evolved starting with small laboratories with 300 W scale up to the present time where the construction of biotechnological power plants is planned since they are low cost, stable, and large energy storage systems; however, it is quite bulky, so more development and more precise control are still required [20].

### 4.5. Thermoelectric and Thermoregulatory Properties of the Oriental Wasp (Vespa Orientalis)

The oriental wasp is the first insect that absorbs solar energy to generate electricity. It has pigments in its tissues that allow it to perform this production, being yellow, the one that traps light, and brown, the one that generates electricity. It is not yet understood how these insects use the generated electrical energy, but it is assumed that the absorbed energy is used in flight and temperature regulation, among others [29]. Studies have been made about the thermoelectric and thermoregulatory properties of the silk produced by the larva of this living being creating a kind of cape. The nest of the oriental wasp is maintained at temperatures of 28 °C while the ambient temperature varies between 20–40 °C. The silk layers help regulate temperatures in the nest by storing excess heat as an electrical charge so that, when the temperature decreases, the energy is released as heat. The wasp nest cocoon consists of fibroin, which is a protein with elastic properties, surrounded by a second protein known as sericin. Together, these make up the silk of the cocoon.

The fibroin core tends to be double-stranded and can be compared to a semiconductor material where the inner strand of this protein performed the function of p (positive) bonds, and the outer sericin envelope performed the function of n (negative) bonds. The closest engineered materials to hornet silk are electrically conductive polymers, such as polyaniline and polythiophane with lodin [30].

To develop an electrical cell capable of generating electricity, storing energy, and releasing it into heat, tests were conducted with the hornet silk layers within a single cell. The silk was obtained from nests in the field. The samples came from the eastern hornet queen with a diameter of 12 mm. They were kept refrigerated until the time of the experimental test. They designed an experimental platform to test the silk samples under varying environmental conditions, including temperature, relative humidity, among others. The tests were performed both in daylight and in the dark [30].

The platform consists of a cylindrical chamber for the control of temperature, relative humidity, light, and darkness. This chamber comprises aluminum with dimensions of 64 mm in diameter, 3 mm thick, and 148 mm high. A 12V DC axial fan at the bottom provides air circulation in the chamber. A 67 W thermoelectric module for cooling control. A 55 W AC radial fan for ventilation airflow and to dissipate heat from the thermoelectric module. Finally, a saline solution container was placed for relative humidity control [30].

As a result, it was obtained that the silk layer presented a voltage of 20 V in 5 s. As soon as the voltage source was turned off, the current was discharged. The capacitance obtained was 21.7 mF. The variation of the resistance along with the temperature had a range of 15.8 MΩ to a minimum of 2.6 MΩ remaining constant in the intervals between 28–35 °C. It was concluded that this is consistent as a material acting as a semiconductor whose performance and functionality depend on temperature and relative humidity. In the dry state, the silk layers act as insulators and, therefore, can be used as capacitors. The proteins that make silk can generate current by applying heat to them; however, moisture plays an important role in electrical transport and converts it from an insulator to a conductor. These fibers are suitable materials for constructing composite walls that could act as electricity generators or capacitors, insulation systems, heat transfer devices, and air filtration systems due to their thermoelectric, thermo-regulating, and storage properties [30].

### 4.6. Sucrose Modification of Li_4_Ti_5_O_12_ Anode Material for Lithium-Ion Batteries

Lithium-ion batteries have made energy storage a broadly developable aspect due to qualities such as high voltage, high energy density, long cycle life, and low pollutants. However, the conventional carbonaceous materials used in the anodes present safety problems because of their low Li intercalation potential, which is close to 0 V. In [31], they improved the electrical conductivity of Li_4_Ti_5_O_12_ (LTO) material through the transport properties of the material with sucrose as a source of organic carbon, thus obtaining a battery with sucrose-modified LTO material with improved electrical conductivity.

**Figure 10:**
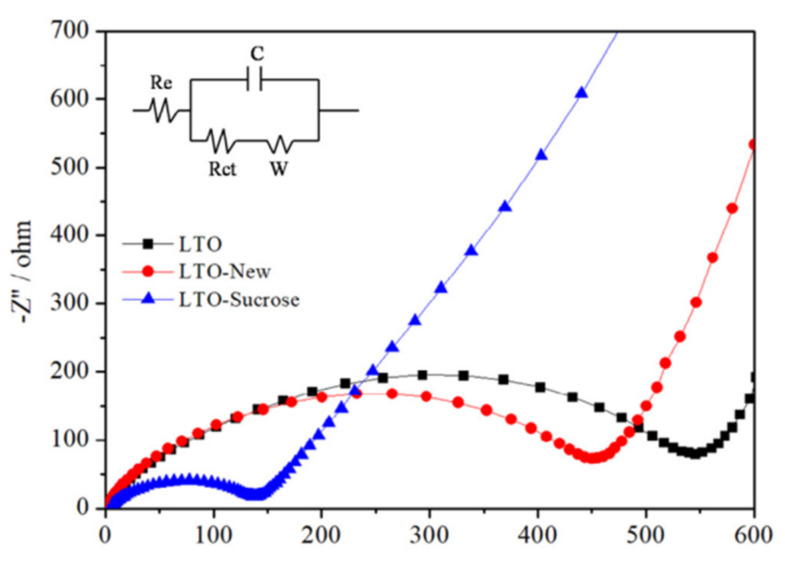
Electrochemical impedance spectroscopy between conventional LTO, LTO manufactured without sucrose, and LTO modified with sucrose [31]. No changes were made to the original figure.

After elaborating the LTO with sucrose, the respective electrochemical impedance spectroscopy measurements at E = 1.55 V were performed. The frequency range was between 0.001–100 kHz under alternating current (AC) stimuli with ten mV amplitude. The experiments were carried out at a room temperature of 25 °C. Figure 10 presents the more detailed spectroscopy analysis where a conventional LTO and the test LTO without sucrose had almost equal resistance to each other and presented three times the resistance of the LTO with sucrose. These results indicated that the modified LTO reduces the charge transfer resistance.

The initial charge–discharge curves of sucrose-modified LTO and LTO samples developed in ranges of 100–200 mA/g showing efficiencies of 90.2% and 92.1%, respectively. The ranges of the charge–discharge voltage platform were 1.7 V and 1.5 V, being close to the theoretical voltage (1.55 V). This was due to their redox reaction. They concluded that using sucrose as a material for LTOs reduces the charge transfer resistance, making it feasible for electron and Li+ transport to benefit charge–discharge cycling.

### 4.7. Improvement of Microbial Cells for Electron Transfer

**Figure 11:**
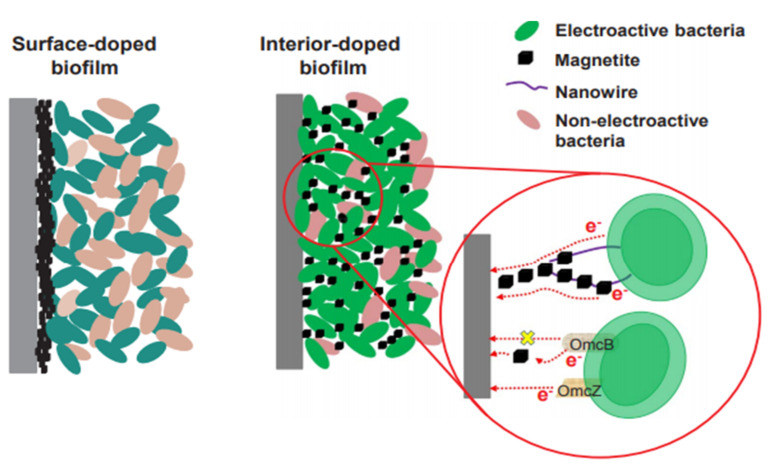
Rough sketch of the study, biofilm doped on the surface (left) and biofilm doped inside (right) [32]. No changes were made to the original figure.

Microbial fuel cells (MFCs) use bacteria as catalysts to convert chemical energy into organic matter and then into electrical energy. These cells are considered green, efficient, and sustainable technology to recover electricity from wastewater treatment. MFCs have been used in many fields such as wastewater treatment, soil remediation, biosensors; however, the low power output of these cells limits their applications. The electron transfer pathways are spatially and mechanically heterogeneous for electroactive bacteria on different parts of the electrode surface. Different materials (quinone, riboflavin) have been used to improve the physicochemical properties of the electrode. These properties correspond to stability and electrical conductivity since both are involved in the electron transfer at the interface between the electroactive bacteria and the electrode. However, as the biofilm grows, most of the electroactive bacteria move away from the electrode surface. Consequently, the electron transfer becomes inefficient, and energy production is limited. This is why, in this study, we use magnetite sprayed on an electroactive biofilm with the help of a magnetic field and also doped a biofilm inside it to facilitate the delivery of electrons from electroactive bacteria away from the electrode surface. Magnetite is a good conductor based on iron oxide for the enhancement of extracellular electron transfer. With the incorporation of magnetite, the electron transfer efficiency improved by 12% and 37%, respectively. The energy density of the MFC doped inside presented results of 764 ± 32 mW, this being a considerable increase with respect to the MFCs with biofilms doped on the surfaces that presented results of 604 ± 22 mW. Figure 11 presents a small scheme of the proposed mechanisms for the simulation of the biofilm doped inside and on its surface [32].

It is worth mentioning that magnetite facilitates the enrichment of electroactive bacteria and helps to increase the proportion of electroactive bacteria to stimulate their production of electrons. Good conductive magnetite allows the collection and transport of more electrons produced by electroactive bacteria even if they are far from the electrode surface. This study demonstrated an effective method for improving bio-electrochemical systems leading to further improvements in the area of batteries and other storage systems to make them less polluting to the environment [32].

### 4.8. Bio-Electrocatalysis for the Production of Green Chemicals, Fuels, and Materials

One way to make bio-based chemicals, biofuels, and biodegradable man-made materials is through bio-electrocatalysis. Such a method can be efficient and more sustainable than conventional methods. It presents an alternative within the area of modern bio-manufacturing technology as it combines biocatalysis and electrocatalysis to produce efficient and green products from electricity. It is important to remember that the redox reaction of biocatalysis requires two substrates (electron donor and electron acceptor) and electron transfer between these substrates.

For microbial cell-based biocatalysis, its diversification in terms of metabolic pathways provides the ability to produce various products. The equivalent residue is able to regenerate through the metabolic activities of the cells. Electrochemical reactions can be used to safely provide redox equivalents for biocatalysis with the consumption of electricity from renewable energy sources such as solar and wind.

Bio-electrocatalysis has been used for the fabrication of biosensors and biofuel cell devices. For this method to be feasible for the preparation of biofuel, chemicals, and other materials, the problem of electron transfer between the electrode surface and the bio-electrocatalyst must be overcome. The bio-electrocatalyst is the main function of the bio-electrocatalysis system and is classified into oxidoreductase and electroactive microbial cell. Oxidoreductases are usually cofactor enzymes with or without metal bases. These oxidoreductases have the advantage of transforming the reduction and oxidation states within the cofactors so that electron transfer is achieved. In contrast, electroactive microbial cells can catalyze a wide range of reactions because the microbial cells act as tiny bioreactors. Other cells have developed the ability to transport electrons as their mechanism to achieve electronic communication between electrodes. The most studied microorganisms for these aspects are “Geobacter sulfurreducens” and “Shewanella oneidensis.”

Currently, better electrode materials are being developed that will allow better performance towards future bioelectrocatalysis devices and systems to be used in the field of chemicals, biofuels, and bioplastics. However, many factors still require further research [33].

**Table 3:** Mechanisms inspired by nature for both storage and generation of electrical energy.

Pinnacle (Strategies)	Mechanism	Principle	Primary Characteristic	Ref.
**Venus Flytraps** It has leaves divided into two movable halves. Once the prey lands inside the open leaves, these halves close, imprisoning the prey inside the plant.	Confine the lignin within the electrode to prevent dissolution by acting as a current collector to provide electron transport during the electrochemical reaction.	Reconfigurable graphene cage to capture lignin.	Environmentally friendly battery electrode materials	2017 [27]
**Photosynthesis** Being an inspired mechanism for electrical energy storage, a strategy (microbial electro-synthesis) capable of absorbing and storing energy from any electrical source is presented.	Use of the modified microbes for long-range electron transport and uptake.	Electroactive microbes are useful for moving hydrogen to microbes which can oxidize it and for a process called SmEET (solid matrix extracellular electron transfer)	Energy storage	2018 [23], 2019 [26]
**Anatomy of leaves**	Based on their structure, dye-sensitized solar cells were used to improve the efficiency of charge collection.	Layer capable of capturing light at the top of the cells (which mimics the epidermis) and photoanodes in the microscale palisaded structure.	Absorption of sunlight	2019 [28]
Plants adapt to any environment, and the orientation of their leaves towards light allows for light-harvesting and photosynthesis.				
***Leptospirillum ferriphilum***	Given the biological oxidation of ferrous ions, they are used as cathode electrons within the electrochemical cell together with hydrogen, which provides the anode electrons.	By utilizing CO_2_ from the atmosphere as the sole source of carbon, they make BioGenerators commercially viable as CO_2_ negative systems.	Power generation	2017 [20]
Iron oxidizing bacteria that have influence as electron collectors for electric power generation in biogenerators.				
**Oriental Hornet (*Vespa Orientalis*)**	Special silk layers of the insect larvae provide thermoelectric and thermoregulatory properties that protect the wasp nest by releasing energy in heat.	The silk created by these insects has fibers suitable for constructing composite walls that could act as electricity generators or capacitors, insulation systems, heat transfer devices, and air filtration systems.	Generation, storage and transfer of energy	2017 [29], 2015 [30]
First organism has the ability to absorb sunlight, store it and then generate electrical energy. Its use of electricity is still unknown.				
**Sucrose**	Comparison between conventional LTO and sucrose modified LTO in which the sucrose modified battery with the abovementioned material provided a reduction in charge transfer resistance, making it feasible for electron and Li+ transport to be beneficial for charge and discharge cycling.	Improvement in the electrical conductivity of Li_4_Ti_5_O_12_ (LTO) material through the transport properties of the material with sucrose as organic carbon source, thus obtaining a battery with sucrose-modified LTO material with improved electrical conductivity.	Anode material for lithium-ion battery electrode and improvement of electrical conductivity	2019 [31]
A proposed material for the improvement and modification of Li_4_Ti_5_O_12_ anodes for lithium-ion batteries.				
**Electroactive Bacteria**	A good conductive magnetite allows the collection and transport of more electrons produced by electroactive bacteria even if they are far from the electrode surface.	Magnetite sprayed on an electroactive biofilm with the help of a magnetic field is used, and a biofilm was also doped inside to facilitate the delivery of electrons from electroactive bacteria away from the electrode surface.	Electron transfer at the interface between the electroactive bacteria and the electrode	2018 [32]
The application of magnetite facilitates the enrichment of electroactive bacteria and helps to increase the proportion of electroactive bacteria to then stimulate their production of electrons.				
***Geobacter sulfurreducens and Shewanella oneidensis***	Electroactive microbial cells can catalyze a wide range of reactions because the microbial cells act as tiny bioreactors.	The redox reaction of biocatalysis requires two substrates (donor and acceptor) and electron transfer between these substrates. Biocatalysis presents a diversification of metabolic pathways and provides the ability to produce a variety of products. The residue is regenerated.	Electron transport and electrode enhancement	2020 [33]
Used in bio-electrocatalysis systems, which is a sustainable way to make certain products as chemicals, biofuels and biodegradable man-made materials.				

## 5. Conclusions

With this study, it was possible to contemplate the evaluation of the different conventional electric energy storage systems detailing advantages and disadvantages of each one, applied to building scale visualizing that currently the most efficient storage system contemplates batteries. As a result of this, alternatives have been sought to improve battery elements, such as hydrogen cells, finding new materials for battery electrodes, among others. Starting from the point of improving elements in these systems, strategies observed in nature or “pinnacles” that are related to the storage or generation of electricity are sought, demonstrating that, although the immediate possibilities of increasing the technical aspects of these systems have not yet been fully investigated, their field of development is quite high, giving way to possible designs of these systems based on biomimetic strategies. Thus, a comprehensive analysis of the available research on biomimicry-based approaches to improving building design, driven by the increasing demands of energy regulations to meet future local goals, has been presented.

Energy storage systems have played a relevant role in applications in different areas, and, for this reason, proposing improvements to these systems continues to be a focus of scientific interest. In this work, it was emphasized that energy storage systems had worked favorably until nowadays, providing great benefits to the consumer or to the applications, but it is necessary to develop environmentally friendly solutions, thus establishing a culture of awareness. Along this line, oriented to the creation of “green” systems with the environment, biomimetic strategies for the development of storage systems have achieved significant advances, ranging from generating energy through the respiratory processes of microorganisms to recreate the generation, storage, and release of energy using the thermoelectric and thermoregulatory characteristics of some insects. These facts show that research and new policies aim to improve existing systems but reinforce the idea that new solutions must be environmentally friendly, so there is still a long way to improve the processes established so far.

## Figures and Tables

**Table 1:** Comparison of characteristics between the different types of batteries according to [13].

Battery Type	Nominal Voltage (V)	Life Cicles (N° of Cicles)	Energy Density (Wh/kg)	Self-Discharge
Lithium-ion (Li-ion)	3.6	500–1000	110–160	Very low
Lead-acid	2	200–300	30–50	Low
Nickel-cadmium (Ni-Cd)	1.25	1500	45–80	Moderate
Nickel-metal hydride (Ni-MH)	1.25	300–500	60–120	High

**Table 2:** Evaluation of conventional storage systems.

Type	Description	Advantages	Disadvantages	Scale of Application	Storage or Generation Capacity	Efficiency	Reference
Hydro-pump	It has two reservoirs or basins connected through tunnels and wells through which water passes from an upper point to a lower one. Inside this system, turbines, pumps, and valves will generate the necessary electrical energy and control the water flow.	High energy storage capacity. Long service life of about 50–100 years.	Location constraints. High capital cost. Low power and energy density. Potential for environmental impacts; however, these systems have been built underground to avoid such impacts.	Buildings with photovoltaic installation for an apartment and villa building, Shanghai (China).	5 kW for both cases (villa, apartment).	76.47% (apartments). 45.13% (villas).	2020 [1], 2018 [3], 2020 [14]
Compressed Air	The air is compressed and accumulated inside a subway reservoir as long as there is excess energy. Then, when an energy demand arises, the accumulated air expands into a turbine which generates electricity.	High energy storage capacity. Decreased energy losses since the system uses substantial fractions for the power supply, which has receivers that reserve the compressed air without the need to use an energy conversion machine.	Geological limitations tend to lead to the installation of steel tanks that can withstand high pressures underground, thus increasing the cost of the system. Variable efficiency. Safety problems. Not suitable for small-scale systems due to the large size of the installations and associated costs.	Residential building with photovoltaic system, Cittá di Castello (Italy).	Pressure 30 bar, generation 1008 kWh. Pressure 225 bar, generation 1273 kWh.	21.9% @30 bar. 26% @225 bar.	2020 [1], 2016 [4], 2018 [15]
Flywheel	The flywheel uses a rotating mass to store energy held in the rotor’s kinetic form. This kinetic energy is transferred in and out of the flywheel with the help of an electrical machine that acts as a generator or motor, depending on whether the system is in charging or discharging mode.	High energy storage capacity. Environmentally friendly. Does not require large space areas. Long service life, depending on the bearings selected (non-contact, non-wearing magnetic bearings tend to have a service life of 20 years).	Noise problems. Safety issues are considered due to high speeds, depending on the robustness of the container. High cost per stored unit. Maintenance. In the long term, the system presents difficulties for energy storage.	Buildings with photovoltaic installation (Germany).	N/D	67% in the short term but decreases to 40% in the long term.	2020 [1], 2018 [5], 2017 [6], 2016 [18]
Lithium Battery	Lithium batteries, including lithium-ion and lithium hydride batteries, represent the most popular battery type among consumer electronic devices due to their low weight, high energy density, and long cycle life.	High efficiency. High power and energy density compared to other batteries. Short response time.	Life cycle depends on discharge levels (5000 cycles (10–15 years)). High cost.	Residential building with photovoltaic installation, Sweden.	7 kWh	85–95%	2020 [1], 2018 [7], 2016 [16], 2016 [18]
Nickel Cadmium Battery	Nickel-cadmium batteries belong to the nickel battery family, including nickel-metal hydride, nickel-iron nickel, and nickel-zinc-based batteries. Inter-cell reactions vary depending on the second component added to the nickel electrode.	Good performance under low temperature. High efficiency. High power density.	High cost.	Commercial buildings, India.	40 MW	70–85%	2020 [1], 2018 [7], 2017 [22]
Lead-Acid Battery	Their cells are based on the reaction between lead, lead oxide, and sulfuric acid. Their cells have a water-based liquid electrolyte that operates at room temperature.	High efficiency but may vary depending on duty cycle and ambient temperature. Low cost.	Low power and energy density. Short response time. Short life cycle (3000 cycles (7–10 years)). High maintenance requirements. High level of toxicity and relatively heavy. Material consumption.	Residential building with photovoltaic system, Cittá di Castello (Italy).	4.6 kWh (total demand of the installation).	70–80%	2020 [1], 2017 [6], 2018 [7], 2018 [15]
Vanadium-Redox Battery	They consist of two electrolyte reservoirs where the electrolyte circulates through an electrochemical cell comprising a cathode, an anode, and a membrane separator. The energy density depends on the stored electrolyte volume and is independent of the electrochemical cell size and design.	High energy storage capacity.	Complex construction. Low energy density.	Residences with photovoltaic installations (Australia)	40–80 kW	75–90%	2020 [1], 2016 [15]
Supercapacitors	It consists of two metal plates separated by a small air gap. The amount of charge accumulated on each plate creates an electric field that balances the charge generated by the voltage.	High energy density. Suitable for high peak power work. Compensate for energy release during short time interruptions.	Cell independency. Safety issues. Life cycle depends on voltage imbalances between cells and maximum voltage thresholds. Environmental impacts.	Power Electronics	8 kWh	95% or higher	2018 [8], 2020 [9]
Magnetic Superconduction	A large superconducting coil has almost no electrical resistance near absolute zero temperature and yet can store electrical energy within the magnetic field generated by direct current flowing through that field.	Immediate response. High efficiency and reliability. Lifetime is independent of duty cycle.	High cost as they have to consider components such as cryogenic vessel, refrigeration, protection, control equipment, coil, and conductor structures. Requires large magnetic fields.	Grid-connected distributed generation structures.	3 MW	95%	2020 [1], 2020 [10]
Hydrogen	It uses excess electricity produced by renewable sources to store it in the form of hydrogen, and when an energy demand arises, the reserved hydrogen is used as a fuel in the power plants.	Low carbon energy so no CO_2_ emissions to the environment. Hydrogen cells satisfy systems during winter seasons. Hydrogen is the lightest element of all.	High initial cost. Hydrogen production is not fully effective.	Building with photovoltaic system (Slovenska Bristica, Slovenia).	N/D	62.13%	2020 [1], 2018, 2017, 2020 [11,12,13]

## Data Availability

Not applicable.

## Abbreviations

BESS	Battery energy storage system
CAES	Compressed air energy storage
DSSC	Dye-sensitized solar cells
EDLC	Electrochemical double-layer supercapacitors
ESS	Energy storage system
FEM	Finite element method
FESS	Flywheel energy storage system
LTO	Li_4_Ti_5_O_12_
MFC	Microbial fuel cells
PEM	Proton exchange membrane
PHES	Pumped-hydro energy storage
PV	Photovoltaic
SMES	Superconducting magnetic energy storage
VRB	Vanadium redox battery
